# Transformation of H-Aggregates and J-Dimers of Water-Soluble Tetrakis (4-carboxyphenyl) Porphyrin in Polyion Complex Micelles

**DOI:** 10.3390/polym10050494

**Published:** 2018-05-03

**Authors:** Shuai Liu, Cun Hu, Ying Wei, Ming Duan, Xin Chen, Yue Hu

**Affiliations:** 1 College of Chemistry and Chemical Engineering, Southwest Petroleum University, Chengdu 610500, China; 201621000202@stu.swpu.edu.cn (C.H.); chenxin830107@pku.edu.cn (X.C.); 201721000233@stu.swpu.edu.cn (Y.H.); 2 Beijing National Laboratory for Molecular Sciences, Institution College of Chemistry and Molecular Engineering, Peking University, Beijing 100871, China; 1601110376@pku.edu.cn

**Keywords:** H-aggregates, J-dimers, transformation, porphyrin, PIC

## Abstract

Tetrakis (4-carboxyphenyl) porphyrin (TCPP) and polyelectrolyte poly(*N*-methyl-2-vinylpyridinium iodide)-*b*-poly(ethylene oxide) (PMVP_41_-*b*-PEO_205_) can self-aggregate into polyion complex (PIC) micelles in alkaline aqueous solution. UV-vis spectroscopy, fluorescence spectroscopy, transmission electron microscope, and dynamic light scattering were carried out to study PIC micelles. Density functional theory (DFT) calculation method was applied to study the interaction of TCPP and PMVP_41_-*b*-PEO_205_. We found that the H-aggregates and J-dimers of anionic TCPP transformed in PIC micelles. H-aggregates of TCPP formed at the charge ratio of TCPP/PMVP_41_-*b*-PEO_205_ 1:2 and J-dimer species at the charge ratio above 1:4, respectively. It is worth noting that the transformation from H-aggregates to J-dimer species of TCPP occurred just by adjusting the ratio of polymer and TCPP rather than by changing other factors such as pH, temperature, and ions.

## 1. Introduction

Water-soluble porphyrins have stimulated tremendous research attention owing to their potential applications in medical science, electronic equipment, biomimetic chemistry, and materials chemistry [1,2,3,4]. Since the relevant applications of water-soluble porphyrins are closely related with their aggregates, great efforts have been devoted to studying the aggregates of water-soluble porphyrins in recent years [5,6,7,8,9]. For example, Shi and co-workers have reported that the dimer and monomer of Fe^III^-tetra(4-sulfonatophenyl)-porphyrin (Fe^III^TPPS) aggregated to form complex micelles when they adjusted the pH value [10]. Tetrakis (4-carboxyphenyl) porphyrin is a well-researched dye due to its ability to self-aggregate into dimer species, J-aggregates (side-by-side arrangement), or H-aggregates (face-to-face arrangement) driven by non-covalently interactions. Some factors, such as a certain pH, changeable ionic strength, different solvents, temperature, and polymer template [11,12,13,14,15,16,17], have been reported to exert significant effects on the transformation of TCPP aggregates.

On the other hand, polyion complex (PIC) micelles have drawn increasing interest in the field of supermolecular self-assembly since they have great potential applications in controlled release [18], transduction of genes [19], delivery of biomolecules [20], nanoreactors [21], and sensors [22]. One of the characteristics of PIC micelles is that they often have core-shell structures, where the micellar core is deeply buried in the forest of the corona [23]. Thus, it is difficult for small molecules to diffuse into the core of micelles. On the contrary, the component that is incorporated in the micellar core is well protected [24]. The aggregates of porphyrin in the core of PIC micelles have also triggered strong interest since they may help further humanity’s understanding of biochemical processes. For instance, Shi et al. studied the aggregation and optical properties of the water-soluble 5, 10, 15, 20-tetrakis (4-sulfonatophenyl) porphyrin (TPPS) in acidic aqueous solution. They found that TPPS still retains the ability to form pH-dependent H- and J-aggregates in the PIC micellar core formed with PEG–P4VP [6]. Porphyrin self-assembly, modulated by pH onto polylysine and a dendrimer template, has been reported [25]. Polystyrene sulfonate–porphyrin assemblies were prepared by Ruthard et al. [26]. They unveiled the influence of linear and cylindrical brushes polystyrene sulfonates and porphyrin structures on the aggregates of porphyrin. However, the interconversion of porphyrin aggregates in PIC micelles just by adjusting the charge ratio of polymer and porphyrin has rarely been reported.

Inspired by these works, we first show the formation of PIC micelles with TCPP and PMVP_41_-*b*-PEO_205_ block co-polymer in alkaline aqueous media, and then report the influence of PIC micelles on the aggregation of TCPP. We report evidence that the TCPP aggregates undergo a transformation as the charge ratio of TCPP and diblock polyelectrolyte increases in an alkaline aqueous solution. The TCPP/PMVP_41_-*b*-PEO_205_ complex micelles were characterized by transmission electron microscope (TEM) and dynamic light scattering (DLS). Formation of J-dimer species and H-aggregates, and the transformation between these two forms were investigated by UV-vis spectroscopy and fluorescence spectra. This is a promising strategy for controlling the aggregates of TCPP in an aqueous solution just by adjusting the polymer concentration and thus may guide the application of TCPP as a fluorescent probe in the self-assembly of aqueous polymer solutions.

## 2. Materials and Methods

### 2.1. Materials

**Scheme 1:**
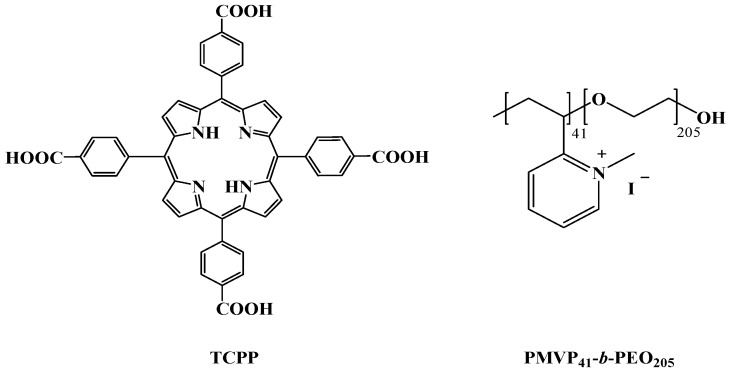
The molecular structures of tetrakis (4-carboxyphenyl) porphyrin (TCPP) and poly(*N*-methyl-2-vinylpyridinium iodide)-*b*-poly(ethylene oxide) (PMVP_41_-*b*-PEO_205_).

Tetrakis (4-carboxyphenyl) porphyrin (TCPP) was purchased from TCI (Shanghai, China). Diblock polyelectrolyte poly(*N*-methyl-2-vinylpyridinium iodide)-*b*-poly(ethylene oxide) (PMVP_41_-*b*-PEO_205_, *M*_w_ = 19 K, PDI = 1.05, about 90% quaternized) used in this work was prepared according to previously reported procedures [27,28,29]. Molecular structures of TCPP and PMVP_41_-*b*-PEO_205_ are shown in Scheme 1. All other chemicals were purchased from Kelong Chem. Reagents Co. (Chengdu, China) and were used without any treatment. Wahaha purified water (Hangzhou, China) was used to prepare solutions and throughout the experiments. The other reagents were of A.R. grade.

### 2.2. Methods

Aqueous solutions of TCPP were initially prepared by dissolving a certain amount of TCPP powder in 0.01 M NaOH aqueous solutions. The desired amount of TCPP solution and PMVP_41_-*b*-PEO_205_ aqueous solutions were fully mixed to obtain a solution with a charge ratio between TCPP and PMVP_41_-*b*-PEO_205_ is 1:0, 1:1, 1:2, 1:5 and 1:8. The charge ratio ƒ is defined as follows: $f=\frac{\left[-\right]}{\left[+\right]}$, where [−] refers to the concentration of negative charges carried by TCPP and [+] refers to the concentration of positive charges carried by the PMVP_41_ block. The final concentration of TCPP is 20 μM, the pH of all above solutions at 10.0 ± 0.2. When the concentration of TCPP is 5 μM, the TCPP/ PMVP_41_-*b*-PEO_205_ solutions were prepared in the same way. All experiments were carried out at room temperature unless otherwise specified.

A UV-1800 spectrophotometer (Shimadzu, Kyoto, Japan) operating in the range of 200~800 nm was used to measure the absorption of solution samples.

A LS55 Fluorescence Spectrometer (PerkinElmer, Waltham, MA, USA) was employed to measure the fluorescence (FL) emission of solution samples. According to the absorption, excitation, and emission spectra (shown in Figure S1), the excitation wavelength was set at 400 nm. Emission spectra were recorded in the range of 570~800 nm.

An ALV/DLS/SLS5022F light-scattering apparatus (Alv-Laser Vertriebsgesellschaft M-B.H., Langen, Germany) was applied to conduct dynamic light scattering (DLS) measurements. The apparatus was equipped with a 22 mW He−Ne laser (632.8 nm wavelength) with a refractive index matching bath of filtered toluene surrounding the cylindrical scattering cell. The samples were filtered through a 450 nm membrane filter. The scattering angle was set at 90°.

A JEOL-100CX II transmission electron microscope (TEM, JEOL, Tokyo, Japan) was used to observe the morphology of the self-assembled micelles. Typically, a drop of sample was placed onto 230 mesh copper grids coated with Formvar film and negatively stained. Then, the sample was allowed to dry naturally for TEM observation.

A steady-state spectrometer FLS920 was applied to determine the decay times of the TCPP/PMVP_41_-*b*-PEO_205_ systems, the excitation wavelength was 400 nm. The fluorescence decays were analyzed using DAS6 software. The experimental time-resolved fluorescence decays can be analyzed using a reported formula [30]:

$$R\left(t\right)=b+\sum_{i}^{n}\alpha_{i}\exp\left(-\frac{t}{\tau_{i}}\right),$$

where *b* is the baseline correction, *n* is the number of discrete emissive species, and *α_i_* and *τ_i_* are the pre-exponential factors and excited-state fluorescence lifetimes associated with the *i*th component, respectively.

The calculations were performed within the spin-unrestricted density functional theory (DFT) framework implemented in the DMol3 code [31,32,33]. The generalized gradient approximation (GGA) with the BLYP functional [34,35], together with double numeric quality basis set (DNP), was used in all calculations. The convergence tolerance for energy change, max displacement, and max force were 2 × 10^−5^ Ha, 0.005 Å, and 0.004 Ha Å^−1^, respectively.

## 3. Results and Discussion

### 3.1. Aggregation Behavior of TCPP at Various Concentrations

**Figure 1:**
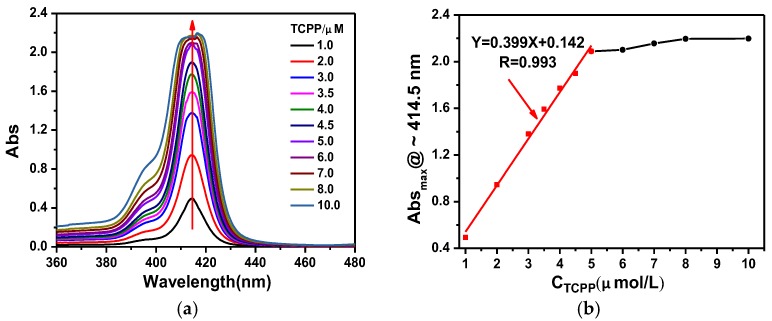
(**a**) UV-vis absorption spectra and (**b**) maximum absorption at ~414.5 nm of TCPP solutions at various concentrations. Red line fits the Lambert–Beer Law, *R* = 0.993. Black line deviates from Beer’s law.

UV-vis spectroscopy was used to measure the critical aggregation concentration of TCPP. Figure 1a shows that the absorption intensity increases as the concentration of TCPP increases. The maximum absorption peak of TCPP is 414.5 nm when the concentration is less than 6 μM. At the range of 1.0~5.0 μM, the intensity of maximum absorption peak increased in a linear fashion, as shown in Figure 1b, which fits the Lambert–Beer Law. In an alkaline aqueous solution and low concentration, TCPP molecules exist in the form of a monomeric free base (TCPP^4−^) due to electrostatic repulsion between TCPP^4−^. Meanwhile, the deviations from Beer’s law at high concentrations (above 5.0 μM) have been well documented and this phenomenon is usually ascribed to the formation of aggregates [36,37,38]. The strongest absorption peak shifted from 414.5 nm to 416.5 nm (Table S1) and split into two peaks when the concentration of TCPP is above 6.0 μM (detailed spectra shown in Figure S2). The slight blue shift of max absorption and its split are considered to be due to the formation of J-dimers rather than J-aggregates under these conditions [39] since the concentration of TCPP > 5 μM is consistent with that previously reported to yield dimerization behavior [40].

### 3.2. PIC Micelles Formed by TCPP and PMVP_41_-b-PEO_205_

**Figure 2:**
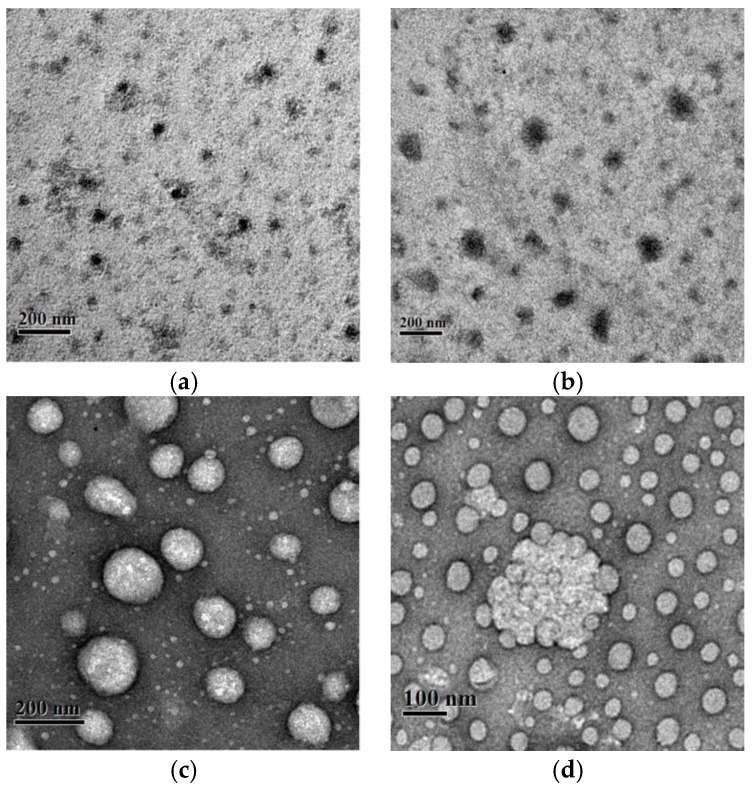
TEM images of polyion complex micelles formed by TCPP and PMVP_41_-*b*-PEO_205_ at the charge ratios of (**a**) 1:1; (**b**) 1:2; (**c**) 1:5 and (**d**) 1:8.

**Figure 3:**
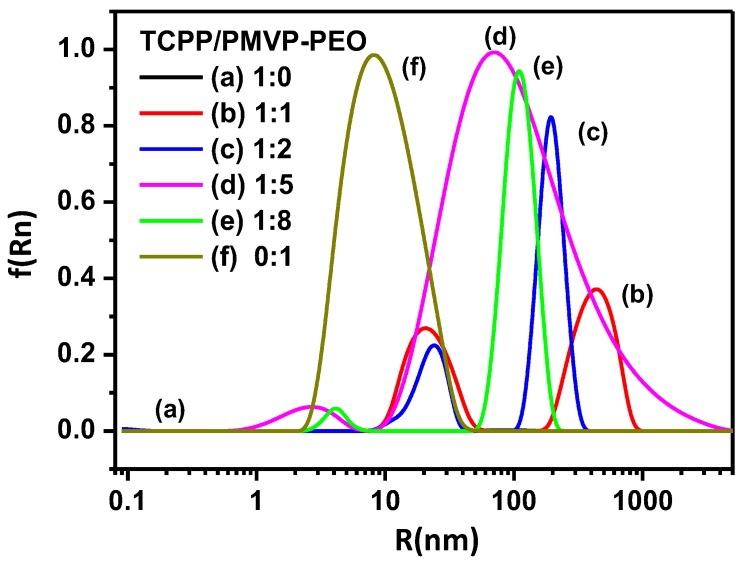
DLS results of polyion complex micelles formed by TCPP and PMVP_41_-*b*-PEO_205_ at the charge ratios of (**a**) 1:0; (**b**) 1:1; (**c**) 1:2; (**d**) 1:5; (**e**) 1:8 and (**f**) 0:1. PMVP-PEO is an abbreviation of PMVP_41_-*b*-PEO_205_.

At a concentration of 20 μM, TCPP molecules mainly exist in the form of J-dimers in alkaline aqueous solution. Upon mixing with the positively charged double hydrophilic block copolymer PMVP_41_-*b*-PEO_205_, a strong Tyndall effect was observed, suggesting the formation of polyion complex micelles (PIC). TEM observations (Figure 2) confirmed the formation of PIC. As can be seen, the average diameter of the micelles are about 20–30 nm, 90–100 nm, 100–130 nm, and 200 nm at charge ratios of TCPP and PMVP_41_-*b*-PEO_205_ of 1:1, 1:2, 1:5, and 1:8, respectively, which are larger than those of micelles (9 nm, Figure 3f) formed with PMVP_41_-*b*-PEO_205_.

In addition, the overall hydrodynamic radius of the micelles was also measured with dynamic light scattering (DLS) as shown in Figure 3. In the absence of polymers, no micelles of TCPP were found, which also proves that TCPP exists in the form of J-dimers instead of J-aggregates. The aggregates of PMVP_41_-*b*-PEO_205_ (20 nm) and TCPP/PMVP_41_*-b*-PEO_205_ (500 nm, not observed under TEM) exist at the same time at the charge ratio is 1:1. As the charge ratio is increased to 1:2, the TCPP and PMVP_41_-*b*-PEO_205_ co-assemble into large aggregates and coexist with the small micelles in the solution. At 1:5, the average hydrodynamic radius of the particles and their polydispersity were observed to strongly increase, which is ascribed to the aggregation of the micelles into large micelles or clusters (Figure 2c,d). For a 1:8 system, the clusters are dominant, which can be observed under TEM, and the dispersion is narrowed.

### 3.3. H-Aggregates and J-Dimers’ Transformation of TCPP in PIC Micelles

**Figure 4:**
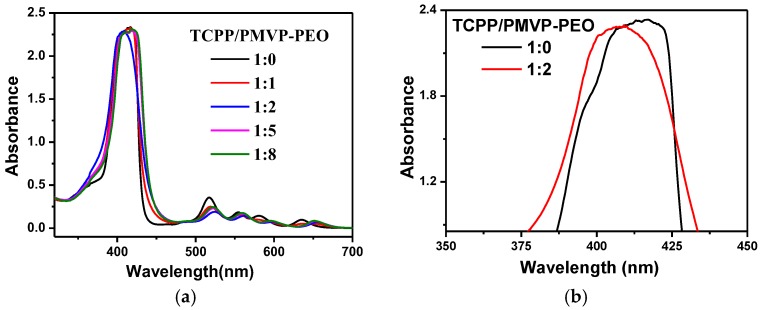
(**a**) UV-vis absorption spectra of TCPP (black line) and TCPP/PMVP-PEO with different charge ratios of 1:0 (black line), 1:1 (red line), 1:2 (blue line), 1:5 (pink line) and 1:8 (green line), respectively; (**b**) Magnified absorption spectra of TCPP and TCPP/PMVP-PEO at the charge ratio of 1:2. [TCPP] = 0.02 mM, pH = 10.0. PMVP-PEO is the abbreviation of PMVP_41_*-b*-PEO_205_.

The formation of PIC micelles leads to a significant change in the UV-vis absorption of TCPP. Figure 4 displays the UV-vis spectrum of TCPP in aqueous solution when it is mixed with the polymer PMVP_41_-*b*-PEO_205_. The black line reveals the characteristic features of TCPP, an intense Soret band at 416.5 nm and four weak Q bands at 517.0, 554.5, 580.5 and 635.0 nm. Upon the formation of micelles, the Soret band of TCPP was blue shifted from 416.5 nm to 406.5 as the charge ratio of TCPP and PMVP_41_-*b*-PEO_205_ at 1:2 and the intensity did not change significantly. The blue shift of sharp and narrow absorption bands indicate the formation of H-aggregates [41]. Moreover, the Q bands appear to exhibit an etio pattern [40] along with decreasing intensities and increasing wavelengths. The red shift of TCPP on the Q band is attributed to the process of TCPP interacting with other components. The red shift of the Q band is caused by the reduction of the energy gap between the highest occupied molecular orbital and the lowest unoccupied molecular orbital [42]. These results suggest that the interaction between TCPP and the polymer in PIC micelles has changed the local environment of TCPP [43].

However, as the concentration of PMVP_41_-*b*-PEO_205_ increased further, the strongest peak moved back to 416.5 nm (Table S2). From this phenomenon, it can be inferred that H-aggregates disappeared and the TCPP molecules exist in the form of J-dimers again. These results are similar to the behaviors in acidic TCPP solutions [44]. The TCPP molecules in the core of PIC micelles exist in the form of H-aggregates at a ratio < 1:4, but still exist in the form of J-dimers at a ratio > 1:4. Moreover, the formation of J-dimers is considered to be due to the formation of PIC micelles rather than ionic strengths, since these experiments are performed at ionic strengths (<50 mM) well below those that correlate with dimerization [45]. In addition, the absorption of TCPP at a concentration of 5 μM was also tested. The absorption spectra are similar to the above results. Detailed data are shown in Figure S3.

**Figure 5:**
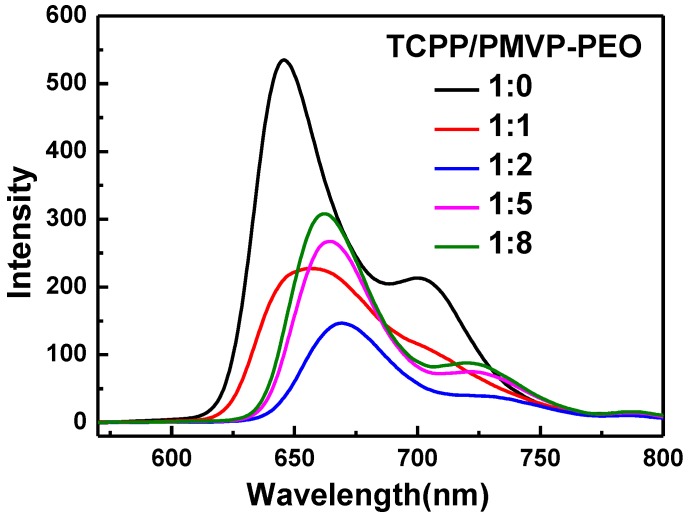
Fluorescence spectra of TCPP (black line) and TCPP/PMVP-PEO with different charge ratios of 1:1 (red line), 1:2 (blue line), 1:5 (pink line) and 1:8 (green line). [TCPP] = 0.02 mM, pH = 10.0, λ_ex_ = 400 nm. PMVP-PEO is an abbreviation of PMVP_41_-*b*-PEO_205_.

The fluorescence spectra of the TCPP and PMVP_41_-*b*-PEO_205_ aggregates at different charge ratios in alkaline aqueous solution are shown in Figure 5. As can be seen, TCPP shows two intense emission bands at 645 nm and 702 nm. With the addition of PMVP_41_-*b*-PEO_205_, fluorescence intensity decreases when the charge ratios are 1:1 and 1:2. At the charge ratio 1:2, the fluorescence intensity is the lowest and the peak position is red-shifted, which is ascribed to the formation of H-aggregates among TCPP. As the charge ratio is greater than 1:2, the fluorescence intensity increases and the peak position exhibits a blue shift. This phenomenon is ascribed to the transformation from H-aggregates to J-dimers of TCPP [46]. The fluorescence spectra are similar to the above results when the concentration of TCPP is 5 μM. Detailed data are shown in Figure S4.

**Figure 6:**
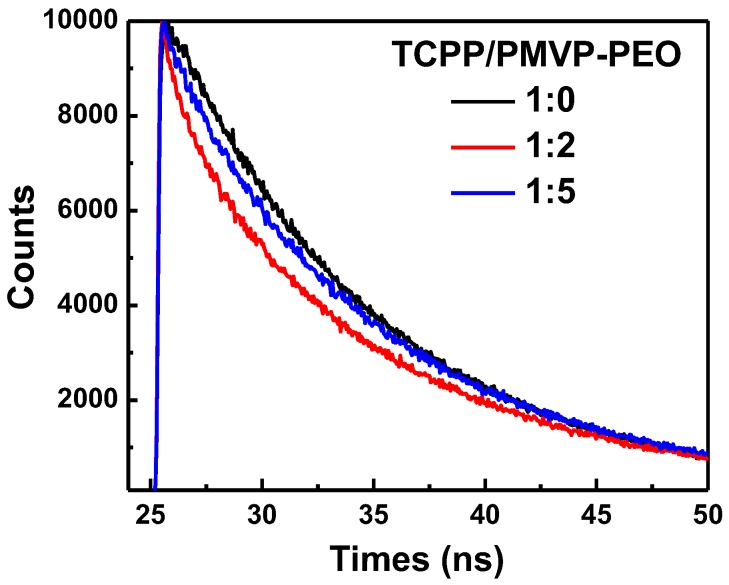
Fluorescence decay time of TCPP and TCPP/PMVP-PEO at different charge ratios of 1:0, 1:2, and 1:5. PMVP-PEO is an abbreviation of PMVP_41_-*b*-PEO_205_.

To understand the aggregates transformation of TCPP in the PIC micelles, we carried out fluorescence lifetime measurements. The fluorescence decay curves of the TCPP/PMVP_41_-*b*–PEO_205_ systems in alkaline aqueous solution are shown in Figure 6. As can be seen, the TCPP afforded a monoexponential fluorescence decay curve with a characteristic lifetime around 9.65 ns. Meanwhile, the other decay curves are fitted biexponentialy. At a charge ratio of 1:2, the decay components are 8.2 ns and 10.3 ns and the average decay time is 9.25 ns. The shortening of decay time of TCPP aggregates from TCPP–dimer unambiguously confirms the H-type aggregation of the TCPP molecules [47]. As the charge ratio reaches 1:5, the decay components are 9.25 ns and 10.2 ns and the average decay time is 9.72 ns. The decay time gradually returned to the dimer state as the concentration of the PMVP_41_-*b*-PEO_205_ increased, which unambiguously confirms the transformation of H-aggregates and J-dimers.

**Figure 7:**
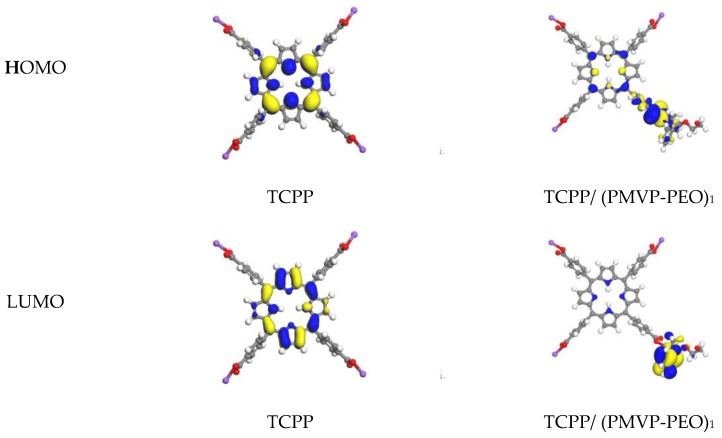
Molecular orbital diagrams calculated for TCPP and TCPP/ (PMVP-PEO)_1_. PMVP-PEO is an abbreviation of PMVP_41_-*b*-PEO_205_.

In order to investigate the influence of PIC micelle formation on the local environment of TCPP, the spin-unrestricted DFT method was employed to understand the effect of PIC on the energy distribution of TCPP. The structure of PMVP_41_-*b*-PEO_205_ was simplified and only one of the monomer units was applied in the calculation, labeled (PMVP-PEO)_1_. Figure 7 shows the HOMO and LUMO of the TCPP before and after interacting with one unit of PMVP_41_-*b*–PEO_205_. As revealed, the energy gap between the HOMO and LUMO of TCPP is 1.76 eV, and it is reduced to 0.85 eV when it interacts with one unit of PMVP_41_-*b*-PEO_205_. This result is supported by the comparison of absorption spectra of TCPP before and after introduction of PMVP_41_-*b*–PEO_205_ (Figure 4). The addition of PMVP_41_-*b*-PEO_205_ induces red shift of the Q band of TCPP.

**Scheme 2:**
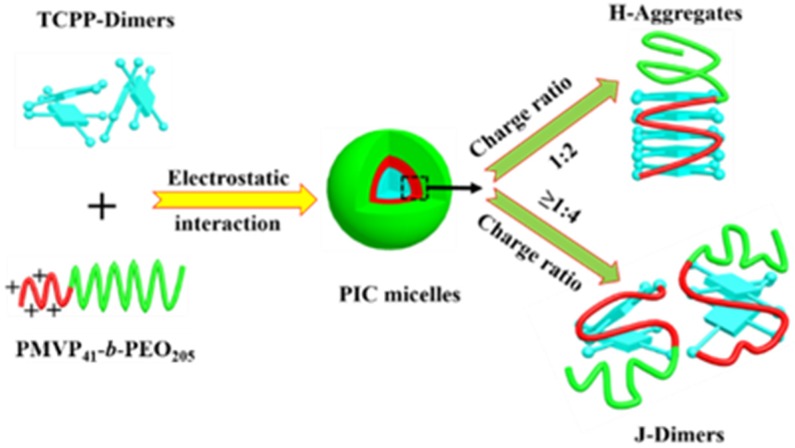
Illustration of the self-assembly of the TCPP/PMVP_41_-*b*–PEO_205_ complex micelles and the transformation of H-aggregates and J-dimers of TCPP in PIC micelles.

With the integration of information indicated by TEM, DLS, UV-vis, FL and DFT analyses, it can be speculated that TCPP and PMVP_41_-*b*-PEO_205_ can self-aggregate into PIC micelles, which might be the reason for the H-aggregates and J-dimers’ transformation of TCPP. The complete scenario for the aggregate transformation of TCPP is graphically depicted in Scheme 2 to facilitate comprehension of this process.

## 4. Conclusions

The transformation from J-dimers to H-aggregates and further to J-dimers represents an interesting model to investigate the assembly, structure, and applications of a wide variety of fluorescent molecules aggregated in PIC micelles. In exploratory experiments, we have shown that a change of various forms can be achieved by adjusting the charge ratio of TCPP and PMVP_41_-*b*-PEO_205_. This result is of particular interest due to there being different advantages of different aggregates ranging from light harvesting capability to catalytic activity. Moreover, the versatility of various aggregates’ formation through controlling the charge ratios of polymer and porphyrins may build a valuable basis for the use of PIC micelles in medicine and catalysis.

## Supplementary Materials

The following are available online at http://www.mdpi.com/2073-4360/10/5/494/s1, Figure S1: Absorption and Fluorescence spectra of TCPP at different excitation. Figure S2: (a) The absorption spectra of TCPP at different concentrations and (b) Magnified absorption peak. Figure S3: UV–vis absorption spectra of TCPP and TCPP/PMVP_41_-*b*-PEO_205_ with different charge ratios. [TCPP] = 5 μM, pH = 10.0. Figure S4: Fluorescence spectra of TCPP and TCPP/PMVP_41_-*b*-PEO_205_ with different charge ratios. [TCPP] = 5 μM, pH = 10.0. Table S1: Maximum absorption peak at various concentrations of TCPP. Table S2: Character absorption peaks of TCPP at different charge ratios of TCPP and PMVP_41_-*b*-PEO_205_.
