# Supplementary material for: Transformation of H-Aggregates and J-Dimers of Water-Soluble Tetrakis (4-carboxyphenyl) Porphyrin in Polyion Complex Micelles
Source: Polymers (Basel). 2018 May 3;10(5):494. doi: 10.3390/polym10050494 (PMC6415385; doi:10.3390/polym10050494)
Supplement: Supplementary file 1 [file polymers-10-00494-s001.pdf]

*Supplementary Materials*

*Article*

# Transformation of H-Aggregates and J-Dimers of Water-Soluble Tetrakis (4-carboxyphenyl) Porphyrin in Polyion Complex Micelles

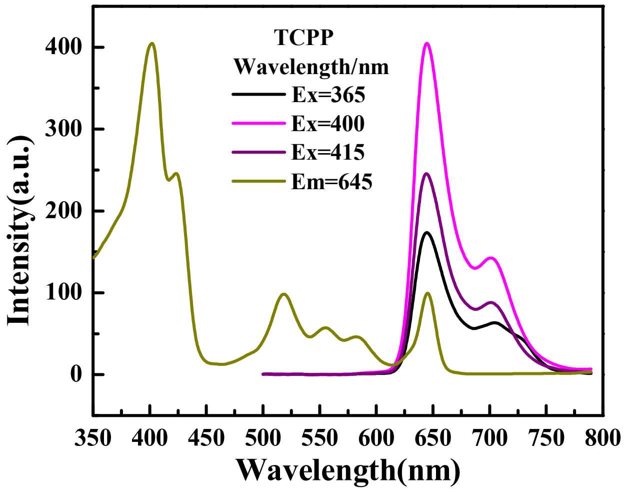


**Figure S1.** Absorption and fluorescence spectra of TCPP at different excitations: 365 nm, 400 nm, and 415 nm. Em = 645 nm. [TCPP] = 5 μM, pH = 10.0.

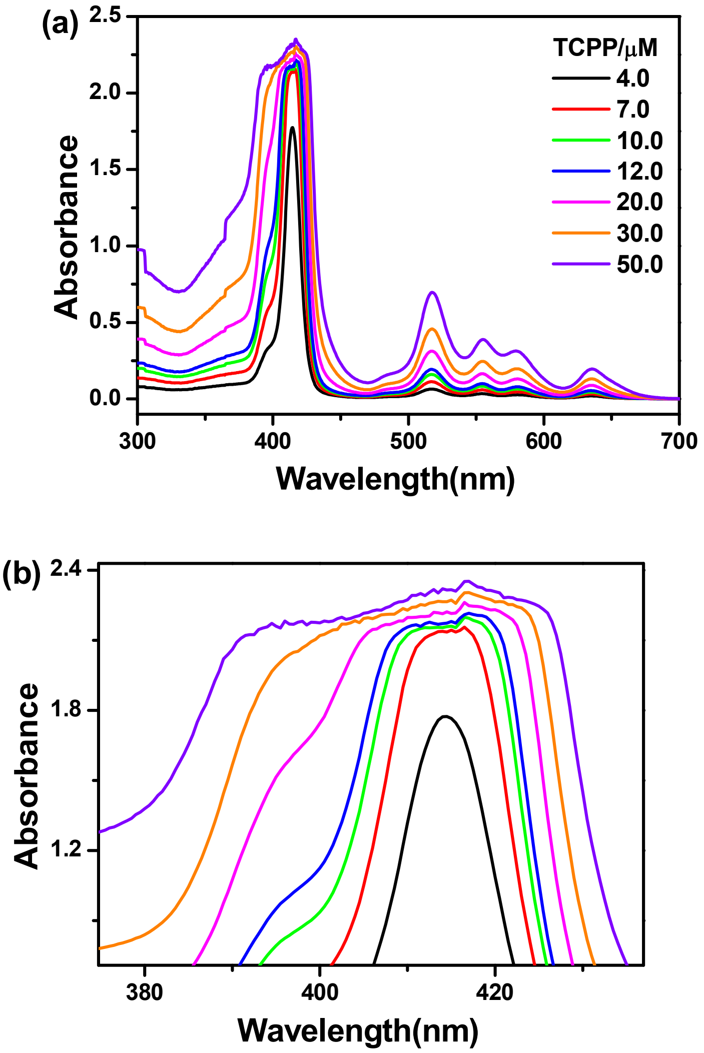


**Figure S2.** (**a**) The absorption spectra of TCPP at different concentrations and (**b**) magnified absorption peak.

**Table S1.** Maximum absorption peak at various concentrations of TCPP.

| System/μM | 4 | 7 | 10 | 12 | 20 | 30 | 50 |
|---|---|---|---|---|---|---|---|
| maximum peak/nm | 414 | 416.5 | 416.5 | 417 | 416.5 | 417 | 417 |

**Table S2.** Character absorption peaks of TCPP at different charge ratios of TCPP and $PMVP_{41}$-*b*-$PEO_{205}$.

| TCPP | | 1:1 | | 1:2 | | 1:3 | | 1:4 | |
|---|---|---|---|---|---|---|---|---|---|
| peak/nm | intensity | peak/nm | intensity | peak/nm | intensity | peak/nm | intensity | peak/nm | intensity |
| 635 | 0.095 | 650 | 0.051 | 654.5 | 0.072 | 654 | 0.076 | 653 | 0.079 |
| 580.5 | 0.141 | 637.5 | 0.053 | 597 | 0.072 | 596 | 0.076 | 596 | 0.08 |
| 554.5 | 0.179 | 556.5 | 0.152 | 561.5 | 0.146 | 560.5 | 0.156 | 560 | 0.166 |
| 517 | 0.346 | 519 | 0.254 | 524 | 0.195 | 523.5 | 0.208 | 523 | 0.223 |
| 416.5 | 2.322 | 416.5 | 2.284 | 406.5 | 2.256 | 408.5 | 2.233 | 416.5 | 2.276 |
| 1:5 | | 1:6 | | 1:7 | | 1:8 | | 1:9 | |
| peak/nm | intensity | peak/nm | intensity | peak/nm | intensity | peak/nm | intensity | peak/nm | intensity |
| 652.5 | 0.085 | 652 | 0.084 | 651.5 | 0.079 | 651.5 | 0.084 | 651 | 0.089 |
| 596 | 0.085 | 596 | 0.085 | 595 | 0.079 | 595 | 0.084 | 595 | 0.089 |
| 559.5 | 0.175 | 559.5 | 0.176 | 559 | 0.171 | 559 | 0.18 | 558.5 | 0.186 |
| 522.5 | 0.237 | 522.5 | 0.24 | 522 | 0.236 | 522 | 0.248 | 522 | 0.254 |
| 416.5 | 2.264 | 416.5 | 2.296 | 416.5 | 2.294 | 417 | 2.294 | 416.5 | 2.298 |

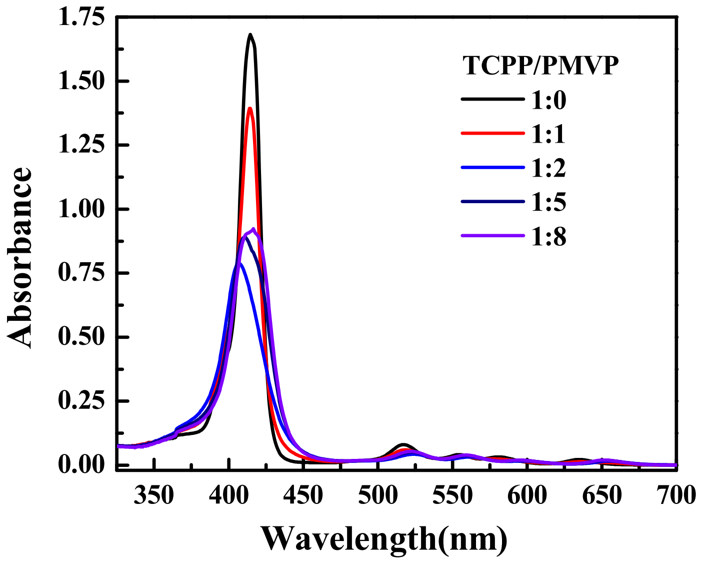


**Figure S3.** UV–vis absorption spectra of TCPP and TCPP/PMVP-PEO at different charge ratios of 1:0, 1:1, 1:2, 1:5, and 1:8. [TCPP] = 5 μM, pH = 10.0. PMVP-PEO is an abbreviation of $PMVP_{41}$-*b*-$PEO_{205}$.

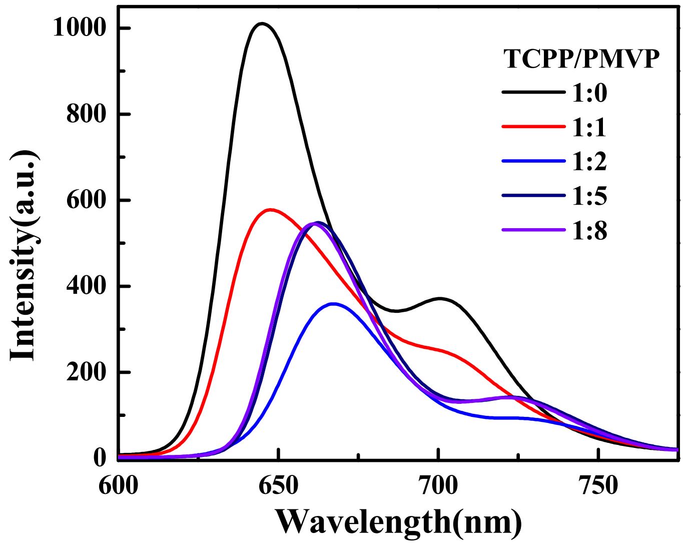


**Figure S4.** Fluorescence spectra of TCPP and TCPP/PMVP-PEO at different charge ratios of 1:0, 1:1, 1:2, 1:5, and 1:8. [TCPP] = 5 μM, pH = 10.0. PMVP-PEO is an abbreviation of $PMVP_{41}$-*b*-$PEO_{205}$.
